# Application of Targeted Next-Generation Sequencing Assay on a Portable Sequencing Platform for Culture-Free Detection of Drug-Resistant Tuberculosis from Clinical Samples

**DOI:** 10.1128/JCM.00632-20

**Published:** 2020-09-22

**Authors:** Andrea M. Cabibbe, Andrea Spitaleri, Simone Battaglia, Rebecca E. Colman, Anita Suresh, Swapna Uplekar, Timothy C. Rodwell, Daniela M. Cirillo

**Affiliations:** a Emerging Bacterial Pathogens Unit, Division of Immunology, Transplantation and Infectious Diseases, IRCCS San Raffaele Scientific Institute, Milan, Italy; b Center for Omics Sciences, IRCCS San Raffaele Scientific Institute, Milan, Italy; c Foundation for Innovative New Diagnostics, Campus Biotech, Geneva, Switzerland; d Department of Medicine, University of California, San Diego, California, USA; Carter BloodCare and Baylor University Medical Center

**Keywords:** direct test, drug-resistant tuberculosis, next-generation sequencing, portable sequencer, rapid drug susceptibility testing

## Abstract

Targeted next-generation sequencing (tNGS) has emerged as a comprehensive alternative to existing methods for drug susceptibility testing (DST) of Mycobacterium tuberculosis from patient sputum samples for clinical diagnosis of drug-resistant tuberculosis (DR-TB). However, the complexity of sequencing platforms has limited their uptake in low-resource settings. The goal of this study was to evaluate the use of the tNGS-based DST solution Genoscreen Deeplex Myc-TB, for use on the compact, low-cost Oxford Nanopore Technologies MinION sequencer. One hundred four DNA samples extracted from smear-positive sputum sediments, previously sequenced using the Deeplex assay on an Illumina MiniSeq, were resequenced on MinION after applying a custom library preparation. MinION read quality, mapping statistics, and variant calling were computed using an in-house pipeline and compared to the reference MiniSeq data. The average percentage of MinION reads mapped to an H37RV reference genome was 90.8%, versus 99.5% on MiniSeq. The mean depths of coverage were 4,151× and 4,177× on MinION and MiniSeq, respectively, with heterogeneous distribution across targeted genes. Composite reference coverage breadth was >99% for both platforms. We observed full concordance between technologies in reporting the clinically relevant drug-resistant markers, including full gene deletions. In conclusion, we demonstrated that the workflow and sequencing data obtained from Deeplex on MinION are comparable to those for the MiniSeq, despite the higher raw error rates on MinION, with the added advantage of MinION’s portability, versatility, and low capital costs. Targeted NGS on MinION is a promising DST solution for rapidly providing clinically relevant data to manage complex DR-TB cases.

## Abstract (precis)

Targeted next-generation sequencing (tNGS) has emerged as a comprehensive alternative to existing methods for drug susceptibility testing (DST) of Mycobacterium tuberculosis from patient sputum samples for clinical diagnosis of drug-resistant tuberculosis (DR-TB). However, the complexity of sequencing platforms has limited their uptake in low-resource settings. The goal of this study was to evaluate the use of the tNGS-based DST solution Genoscreen Deeplex Myc-TB, for use on the compact, low-cost Oxford Nanopore Technologies MinION sequencer.

## INTRODUCTION

The World Health Organization’s (WHO) End TB Strategy calls for universal access to comprehensive drug susceptibility testing (DST) and early diagnosis of all persons with any form of tuberculosis (TB), as a priority and key component of integrated, patient-centered TB care (1). Despite global investments in improving laboratory capacity, in 2018, only 51% of people with bacteriologically confirmed TB were tested for rifampicin resistance (RIF^r^) and less than 60% of the notified multidrug-resistant (MDR)/RIF^r^ patients were tested for second-line resistance (2). The long turnaround time, the relatively low throughput, and demanding laboratory biosafety requirements make the culture-based phenotypic DST unsuitable as primary tool for clinical management of complex TB cases (3).

The uptake of WHO-recommended rapid diagnostics (WRDs), Xpert MTB/RIF Ultra (Cepheid, Sunnyvale, CA), and line probe assays for use on direct clinical samples has contributed significantly to the global increase in number of RIF^r^ TB cases detected and has reduced diagnostic and treatment delays (4–6). However, the current WRDs are designed to interrogate only short genomic regions harboring the high-prevalence mutations (i.e., “hot spots”) associated with phenotypic resistance to most, but not all, of the first- and second-line drugs. In addition, current WRDs cannot fully discriminate mutations conferring low- and high-level resistance, or mutations not associated with resistance (7), and cannot be adapted easily to detect new genomic regions associated with resistance to novel and repurposed TB medications recently approved for treatment by the WHO (8).

Next-generation sequencing (NGS) of Mycobacterium tuberculosis complex (MTBC) can provide deep and comprehensive data on all clinically relevant resistance mutations and is the leading alternative to existing phenotypic and molecular DST methods (9) currently used for diagnosing drug-resistant TB (DR-TB). The effective implementation of whole-genome sequencing (WGS) in clinical settings, however, is currently limited by the need for an initial TB culture step to generate a sufficient bacterial load for successful sequencing (10).

In contrast, targeted NGS (tNGS) can be performed on direct clinical samples and has emerged as a feasible option for comprehensive, fast, and clinically relevant sequencing from patient sputum samples (11–15). However, the complexity, cost, and laboratory requirements of many sequencing platforms have limited the broad uptake of culture-free tNGS for DST in the routine, low-resource settings that carry the highest diagnostic burden. The use of novel, nanopore-based DNA sequencing instruments (e.g., MinION; Oxford Nanopore Technologies [ONT], Oxford, UK) in low-resource and diverse field settings has emphasized the added value of these instruments (16) as clinical tools. They are portable, robust, and low-capital-cost sequencers that could conceivably be utilized in near-patient settings to conduct tNGS in a manner that could transform TB DST and the treatment decision making process (17, 18). Indeed, the Minion platform has the potential to penetrate clinical laboratories far more broadly than other sequencing solutions in terms of cost, space, maintenance, and clinical utility, with the added value of potentially being useful for identifying resistance-conferring mutations in near real-time (19, 20). In addition, large-scale implementation of these portable sequencers could provide early information on transmission through strain identification, resulting in more efficient contact tracing and epidemiological investigations.

The aim of this study was to evaluate the adaptability of an existing TB tNGS assay kit and DST analytics solution (Deeplex Myc-TB; Genoscreen, Lille, France), originally developed for application on Illumina (San Diego, CA) sequencing instruments, for use on the portable ONT MinION sequencer in order to demonstrate the feasibility and potential of rapid tNGS on a compact, low-cost sequencer.

## MATERIALS AND METHODS

### Sample collection.

For this study, 104 DNA samples from randomly selected smear-positive, Xpert MTB/RIF-positive sputum specimens, previously characterized by Deeplex Myc-TB on the Illumina MiniSeq platform, were resequenced using MinION. Samples were retrieved from the archived collection available at Ospedale San Raffaele, Milan, Italy. No patient data were used, and the study was done without any modification of clinical and laboratory management.

### Sample preparation.

We performed ONT MinION sequencing on the Deeplex Myc-TB amplicons produced from clinical samples previously sequenced on the Illumina MiniSeq instrument, in order to compare and contrast results and performance. Sequencing of Deeplex Myc-TB amplicons on MiniSeq was considered the reference technology for this study. Laboratory procedures, including sputum sample processing, DNA extraction and purification, and PCR amplification by the Deeplex Myc-TB kit, are described in detail in the supplemental material.

### Library preparation and sequencing.

While the sample preparation steps were common to both sequencing instruments, the library preparation steps for sequencing on the MinION and MiniSeq were different based on the manufacturers’ instructions. Each previously produced Deeplex Myc-TB hi-plex amplicon was used for MinION library preparation, as described in detail in the supplemental material.

### Postsequencing analysis pipelines.

The Deeplex Myc-TB kit contains a sequencing analysis and clinical interpretation software solution originally developed for Illumina raw sequence data. This solution is currently not optimized for sequence data from the MinION. For the purposes of this study, we therefore computed read quality and mapping statistics for MinION data using an in-house, proprietary pipeline we developed, while for the MiniSeq results we used the software solution provided by the manufacturer as described in the supplemental material and complemented the analysis with the MTBseq pipeline (21). The read quality, mapping statistics, and overall number of nucleotide variants (i.e., any single nucleotide polymorphism [SNP] or nucleotide insertion/deletion [indel]) identified by MinION and MiniSeq were then compared. The pipelines adopted are described in detail in the supplemental material. Mutations associated with drug resistance were determined according to standardized methods previously published (22, 23).

## RESULTS

All 104 sediment samples amplified by Deeplex Myc-TB and sequenced using MiniSeq were also successfully sequenced on MinION.

### MiniSeq mapping statistics.

**FIG 1:**
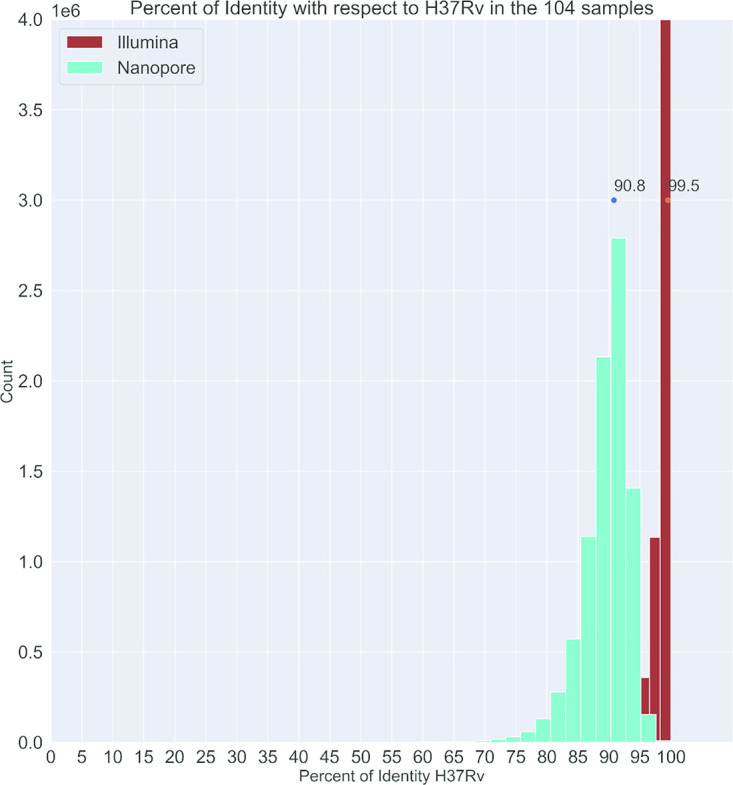
Histogram of percentage of reads identity with respect to Mycobacterium tuberculosis H37Rv for MinION (green bars) and MiniSeq (brown bars). The dots are the maximum value (90.8% and 99.5%, respectively).

**FIG 2:**
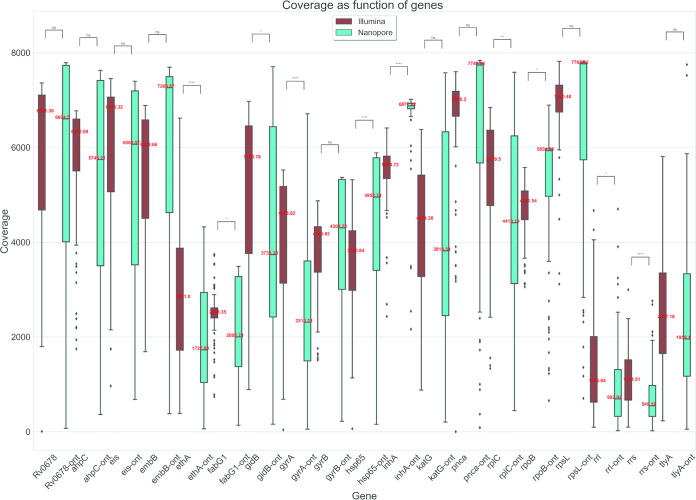
Coverage depth as function of genes for MinION (ONT) and MiniSeq, with green and brown for each gene, respectively. Mean values are reported in red. *P* values are indicated as follows: ns (not significant), 5.00e−02 < *P* ≤ 1.00e + 00; *, 1.00e−02 < *P* ≤ 5.00e−02; **, 1.00e−03 < *P* ≤ 1.00e−02; ***, 1.00e−04 <*P* ≤ 1.00e−03; ****, *P* ≤ 1.00e−04.

The average percentage of MiniSeq reads identity mapping to the reference genome was 99.5% (Fig. 1). The average depth of sequencing coverage obtained on MiniSeq was 4,177×. Mean composite reference coverage breadth of the study samples was over 99%, indicating complete coverage of the targeted genome regions (Data Set S2). The MiniSeq coverage depth was stratified by targeted genomic region for all 104 samples (Fig. 2 and Data Set S3).

### MinION mapping statistics.

Two sequencing runs of 48 and 56 samples were performed on the MinION device using two flow cells to sequence the whole study collection of 104 isolates. The average identity percentage of MinION reads mapping to the H37RV targeted genome was 90.8%, with a MAPQ (MAPping Quality) value around 60 (Fig. 1).

The average depth of sequencing coverage relative to reference genome obtained on MinION was 4,151×. As with MiniSeq, the MinION coverage depth was stratified by targeted genomic region for all study samples (Data Sets S4 and S5). Figure 2 shows a heterogeneous coverage depth distribution across genes associated with drug resistance, and lower coverage in the ribosomal region (*rrs* and *rrl*). Mean composite reference coverage breadth was over 99%, indicating complete coverage of the targeted genome regions (Data Sets S6 and S7).

### Variant calling: MinION versus MiniSeq.

**FIG 3:**
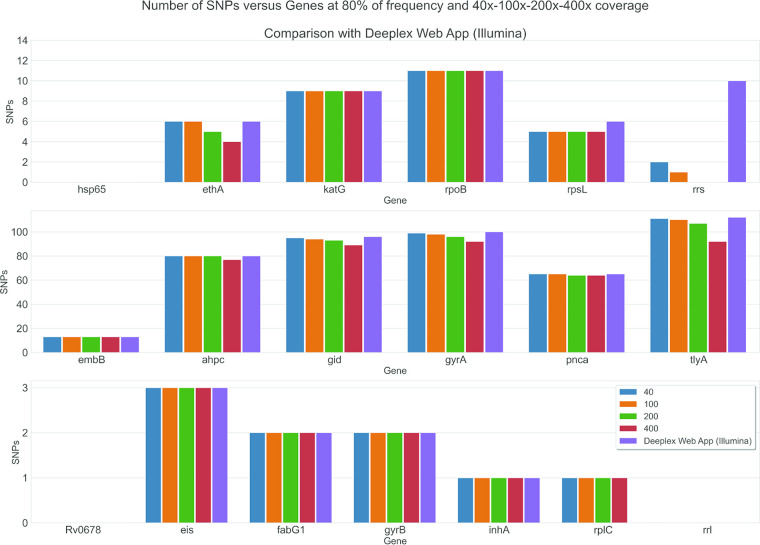
Number of SNPs as function of genes. The purple bar represents the MiniSeq data analyzed by Deeplex Myc-TB web application at default 200× depth coverage minimum.

**FIG 4:**
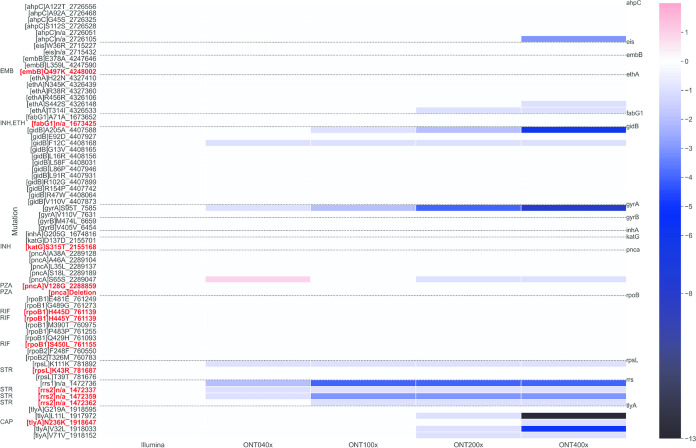
Heat map displaying the number of SNPs found with MinION and MiniSeq approaches as function of the coverage depth. The MinION SNP calls are determined at 80% frequency. MiniSeq data were analyzed with the Deeplex Myc-TB web application at 200× threshold (default setting). The dotted horizontal lines outline the targeted genomic regions and SNPs found within them. On the left, SNPs found in both technologies for at least one sample are shown. The color map goes from +1 to −13, where the values are from the difference between (SNPs [MinION] – SNPs [MiniSeq]) along the 104 samples. The value of 0 means agreement between MinION and MiniSeq technologies (white). Mutations associated with drug resistance are reported in red, and the drug is specified on the left.

We compared the total variant calls between the MinION and MiniSeq, setting different minimum coverage thresholds to explore variant calling with MinION, as described in the supplemental material. Overall, we observed good overlap of variant calls between sequences generated from MinION and MiniSeq (Fig. 3 and Data Set S17; Fig. 4 and Data Set S18). No substantial changes in MinION variant calls were found as a result of minimum coverage values considered. Discrepant total SNP calls were found on MinION compared to MiniSeq when stringent (i.e., 400×) minimum cutoffs were applied, resulting in fewer calls from MinION.

Regarding the presence of confidence-graded mutations associated with resistance (22, 23) to the main drugs, we observed full concordance between MinION and MiniSeq results when we fixed minimum coverage thresholds to call a variant on MinION to 40× (Fig. 4). Only two resistance-associated mutations in the *rrs* gene (linked to streptomycin resistance at genomic coordinates 1472337 and 1572359) were detected by MiniSeq and missed by MinION at the 40× threshold adopted, but these were, however, identified also by MinION with a lower number of reads (10 and 38 reads, respectively [Data Sets S8 and S12]). The MinION method correctly identified the resistance patterns determined by MiniSeq, including 3 RIF^r^, isoniazid-resistant (INH^r^) strains with additional pyrazinamide resistance (PZA^r^) and streptomycin resistance (SM^r^), PZA^r^ and ethambutol resistance (EMB^r^), and PZA^r^ only; 1 RIF^r^, INH-susceptible (INH^s^) sample with additional capreomycin resistance (CAP^r^); 6 RIF^s^, INH^r^ cases, with additional SM^r^ in 3 cases; and 5 strains showing SM^r^ only (Data Set S19). In two samples we observed a full *pncA* gene deletion on MiniSeq, which was correctly identified by MinION as well.

## DISCUSSION

Replacing or complementing phenotypic DST with tNGS-based resistance prediction could avoid or significantly decrease our reliance on culture-based DST methods and has the potential to transform the management of DR-TB globally. The recent development of compact, portable NGS instruments for field deployment will accelerate the decentralization of NGS testing and make near-patient tNGS for clinical diagnosis of drug-resistant TB a near-term reality, with remarkable time and cost savings compared to conventional sequencing instruments (16, 18). The early availability of an accurate molecular profile of the TB strain infecting a patient will enable health care workers to establish evidence-based treatments at the time of diagnosis, which will minimize the risk of inadequate treatment regimens being started. Additionally, for drugs such as ethambutol and pyrazinamide, for which phenotypic DST remains less reproducible than molecular prediction of resistance (24), tNGS is an added-value clinical tool.

In this study, we demonstrated that the existing tNGS Deeplex Myc-TB assay, optimized primarily for larger Illumina sequencing platforms, could be successfully implemented on a portable MinION sequencing device. With only minor protocol modifications, we were able to adapt the library preparation of amplicons (average size of 600 bp) for optimal, long-read sequencing direct from patient clinical samples. This approach resulted in excellent coverage across the targeted MTBC genomic regions, but with higher error rates than for MiniSeq sequences from the same sample amplicons (Fig. 1). Critical to the success of this adaptation was the flexible bioinformatics pipeline we developed, which allowed us to set optimal minimum coverage thresholds to compensate for the error rates in MinION data and enabled us to detect all clinically relevant variants also identified by the MiniSeq instrument and standard bioinformatics pipeline.

The raw base call error rate was found to be high in MinION sequencing, at around 9%, in comparison to the negligible error rates in MiniSeq data (Fig. 1). Even accepting that around 0.5% of identified mismatches compared to the reference genome are actually polymorphisms, in line with the mismatch percentage shown by Illumina, where more than 90% of reads, on average, had extremely high base call accuracy (99.9%), the average identity to the reference genome remained quite low for the MinION data. The 9% rate is in line with those described in previous publications on nanopore 1D read sequencing (17, 25). The deep coverage we obtained on the MinION, an intrinsic feature of tNGS, however, enabled us to still generate enough high-quality reads to identify all of the clinically relevant variants accurately. Furthermore, the downstream bioinformatics analysis pipeline that we tailored for MinION data enabled us to remove the low-quality reads, which helped us minimize false-positive calls.

The variable MinION depth of coverage for the Deeplex Myc-TB-targeted regions (Fig. 2) was another source of complexity in our analyses, requiring a focus on higher coverage values on the runs to obtain interpretable results from all the gene regions interrogated. This heterogeneous distribution was also observed in the MiniSeq data generated from the same amplicons, although with narrower interquartile ranges and closer outliers (boxplot in Fig. 2), and can be attributed to the different sizes of fragments, different PCR amplification efficiencies, differences in GC content affecting the sequencing quality, and the presence of contaminant DNA material in the direct samples. All of these components likely affected the quality of the reads generated (particularly true for the ribosomal region conserved among bacteria, *rrs* and *rrl*).

With regard to variant calling, we have demonstrated that by applying appropriate minimum call thresholds to the coverage depth at a given target region (Fig. 3 and 4), it is possible to determine with high confidence the presence of a true variant allele in a clinical sample sequenced on MinION that is present at a frequency of ≥80%.

The majority of discrepant variant calls between MiniSeq and MinION were found when the stringent 400× minimum cutoff to call a variant (red bar in Fig. 3) was applied to MinION data. This was especially true for *rrs*, *tlyA*, *gidB*, and *gyrA* (Fig. 4) and led to indeterminate results when a given position did not reach the minimum coverage needed to make a call. This was also evident in gene regions showing lower coverage depths, on average (Fig. 2; see, e.g., *rrs* and *tlyA*).

Concerning the detection of resistance-conferring mutations, the two technologies produced almost identical variant calls when the analyses were performed at MinION 40× minimum coverage (Fig. 3 and 4 and supplemental material) and MiniSeq at Deeplex Myc-TB web application default settings. Interestingly, two full *pncA* deletions were correctly detected by MinION using the Porechop demultiplexing approach using the MiniSeq sequencing data from the same amplicons as a reference. Conversely, the Albacore and Guppy software aligned a small number of reads to the *pncA* region of these two samples, i.e., 40 to 60, likely due to the misclassification barcoding process, in which reads were assigned to wrong sample (25). This disagreement between Albacore/Guppy and Porechop is due to the very stringent settings applied by Porechop, which result in more reads in the unclassified bin but lower risk of misclassification. This condition should reduce the risk of cross-barcode contamination and, therefore, the false-positive detection of indels and SNPs by MinION, which is essential for clinical use cases.

The costs to run Deeplex Myc-TB on MinION and MiniSeq, considering kits, reagents and consumables for DNA extraction, targeted amplification, and sequencing at the maximum multiplexing achievable with the two platforms, were comparable and in the range of €100 per sample. The targeted depths of sequencing coverage were similar for the two platforms (4000×), and batch size for a single run was higher on MiniSeq than on MinION (72 versus 56 on MiniSeq and MinION, respectively). In this study, we assessed two different batch sizes for MinION (48 and 56) obtaining linear cumulative yield in gigabases, suggesting that MinION throughput may be still increased over 56, without significant loss in coverage depth.

Turnaround times to prepare samples for MinION and MiniSeq sequencing reactions from DNA extraction were also similar (3 working days). Future customization of Deeplex Myc-TB primers for MinION chemistry, avoiding the need of library preparation modifications to the protocol, would enable shorter library preparation time and is something that should be explored. Importantly, the length of the MinION run could also be optimized to reduce time and cost. In the two study runs, the cumulative yield of reads after 12 h accounted for about the 60% of the total (Data set S20), with read lengths constant up to the end of the MinION sequencing. Therefore, the two runs could have been stopped after 12 h with a >2,000× average depth of sequencing coverage from the samples included in the run, likely enabling one to reliably analyze all Deeplex Myc-TB targets. Batching fewer samples on a MinION amplicon run, likely the pragmatic solution for a routine laboratory with limited daily samples, would enable one to generate optimal sequencing reads for clinical interpretation within a few hours. Additionally, there is the potential to reuse flow cells on MinION, which is not possible on MiniSeq and could significantly reduce the time and cost per sample sequenced. The versatility and physical robustness of the MinION compared to MiniSeq, accompanied by lower capital and maintenance costs and the demonstrated accuracy for identifying clinically relevant mutations in MTBC, make this tNGS platform a promising solution for decentralized, culture-free clinical sequencing in laboratories with fewer daily samples.

This study has some limitations. The Deeplex Myc-TB assay was applied only on smear-positive sputum samples, and prior complete sequencing results were obtained from all selected samples on the Illumina platform. The sensitivity and specificity of this tNGS solution (Deeplex Myc-TB and MinION sequencing) still need to be evaluated using appropriate analytical and clinical studies. Mycobacterial species identification and MTBC genotyping were not assessed with MinION data, as the current study was meant primarily to compare the total variant calls and drug resistance interpretation of the two sequencing technologies.

The high-frequency-variant threshold adopted for MinION (i.e., 80% in this analysis) was set to avoid the introduction of additional variables in this first mutation analysis comparison between the two platforms. Furthermore, the Deeplex web application used to analyze the MiniSeq data did not detect the presence of any relevant low-frequency variant (at 3% threshold); therefore, the overall comparative SNP analysis should not be biased.

In this study, we demonstrated that the workflow and sequencing data obtained from Deeplex Myc-TB amplicons from direct clinical specimens, sequenced on a MinION instrument, are comparable to the Illumina MiniSeq process and outputs, with the added advantage of the MinION device’s portability and low capital costs, making this platform more suitable for use in clinical mycobacteriology and to a wider extent in clinical microbiology laboratories where the same platform can be used to identify MDR bacterial pathogens (20). The application of tNGS on portable, solid-state sequencing devices holds enormous promise for precision global health in the management of DR-TB, where sequencing data obtained from direct clinical samples could inform clinical decision systems supporting individual treatment decisions and large-scale programmatic molecular DST (26). More broadly, portable and compact sequencing technologies are demonstrating their valuable role in the fight against emerging infectious diseases (27).

## Supplementary Material

Supplemental file 1

Supplemental file 2
