# Supplementary material for: Application of Targeted Next-Generation Sequencing Assay on a Portable Sequencing Platform for Culture-Free Detection of Drug-Resistant Tuberculosis from Clinical Samples
Source: J Clin Microbiol. 2020 Sep 22;58(10):e00632-20. doi: 10.1128/JCM.00632-20 (PMC7512157; doi:10.1128/JCM.00632-20)

## Supplementary Material

### Methods

**Sample preparation:** Sputum samples were liquefied and decontaminated using OMNIgene·SPUTUM kit (DNA Genotek, Ottawa, Canada) as previously described (1), and transported to the sequencing laboratory. The specimens were then centrifuged, and genomic DNA was extracted from the resuspended sediments by heat and enzymatic lysis. Briefly, 200 µL of sediment were heat-inactivated at 95°C for 30 minutes, and lysed using 20 µl of Proteinase K solution (20 mg/mL) and 130 µL of incubation buffer (provided with the Maxwell 16 FFPE Tissue LEV DNA Purification Kit, Promega, Madison, WI, USA) at 60°C. Four hundred µl of Lysis buffer (provided with the kit) were added to the sample and the tube vortexed. The sample was transferred to the Maxwell 16 LEV cartridge for DNA purification on Maxwell 16 MDx LEV device according to manufacturer's instructions, with final DNA resuspension in 25 µL of molecular-grade water.

**PCR amplification for tNGS:** The obtained DNA samples were subjected to a 24-plexed PCR using a single Master Mix to amplify phylogenetically informative and resistance-associated MTBC gene targets using the Deeplex Myc-TB Kit (Beta version), according to manufacturer's instructions. The assay amplifies 18 main DR-associated MTBC gene targets, enabling resistance prediction to 13 anti-TB drugs/drug classes, including first-, second-line drugs and compounds of recent introduction, such as bedaquiline and linezolid (2, 3). The amplicons obtained were purified using paramagnetic AMPure XP beads (Beckman Coulter, Brea, CA, USA) and quantified on the Qubit 3.0 Fluoremeter (Thermo Fisher Scientific, Waltham, MA, USA) by using Qubit dsDNA HS Assay Kit.

**Library preparation and sequencing:**

*MinION*

The Deeplex Myc-TB amplicons were prepared for run on MinION with Flow Cell R9.4 (up to 512 nanopore channels) using a modified 1D PCR barcoding (96) genomic DNA (SQK-LSK108) protocol. One of the most significant complexities of using a long-read sequencer (MinION) for sequencing short amplicons (optimal for technologies generating short reads, such as Illumina) is that the multiplex amplicons were too short to be optimally processed by the MinION. Indeed, the average size of Deeplex Myc-TB amplicons considered in the study is around 600 base pairs, with the longest PCR product of over 1500 base pairs and shortest amplicons in the range of 400 base pairs. The MinION library preparation protocol was altered to fit the long reads nature of MinION chemistry to the short size of Deeplex Myc-TB amplicons. Briefly, the double-stranded DNA amplicons were end-repaired and dA-tailed for the adapter ligation step by extending the reaction incubation to 30 minutes at 20°C and 30 minutes at 65°C, instead of 5 minutes and 5 minutes, respectively. After ligation of barcode adapters, libraries were purified with AMPure XP beads at concentration 0,7x, instead of 0,4x, enabling purification of only amplicons ≥200 base pair in length. The barcoding PCR was set up with the recommended cycling conditions but extending the number of PCR cycles to 18 and adjusting the extension time to 90 seconds each cycle. Forty-eight and 56 barcoded samples were pooled to obtained ~ 1µg barcoded libraries for the two sequencing runs, respectively, and the second end-prep incubation was performed at the conditions described above. As library fragment mean size was less than 1500 base pairs, 0.2pmoles of the pool was used in the adapter ligation step. The adapted DNA library pools were purified with AMPure XP beads at concentration 0,7x before loading into the flow cell. MinION run time was set at 24 hours.

*Miniseq*

The Deeplex Myc-TB amplicons were prepared for run on Illumina MiniSeq platform with the MiniSeq High Output kit (300 cycles) using the standard Nextera XT DNA Library Prep Kit (Illumina Inc, San Diego, CA, USA) to obtain paired-end libraries (2). Pools of 9, 24, 28, and 43 barcoded samples were created for 4 runs on MiniSeq, respectively. The length of each run on the MiniSeq platform was 24 hours.

**Post-sequencing analysis pipelines:**

*MinION*

An *in-house* high-throughput data analysis bioinformatics pipeline was used to analyze the long reads from the MinION technology. The *FAST5* reads were basecalled with Albacore (v2.3.1). The resulting *FASTQ* files were demultiplexed with different tools, including Albacore, Guppy (v3.0.3+7e7b7d0) and Porechop (https://github.com/rrwick/Porechop v0.2.4) (4), in order to reduce cross-barcode contamination. Different settings for each tool were exploited to get reliable barcoded *FASTQ file.* The resulting best tool was Porechop with *--barcode_threshold 85 --discard_middle –require_two_barcodes* options. The reads were mapped to H37Rv *Mycobacterium tuberculosis* reference strain (GenBank NC_000962.3) with Minimap2 (v.2.15-r905) (5) with the options *-ax map-ont –L* and converted to a sorted BAM file using Samtools (v1.2). Per-nucleotide depth coverage and genome breadth per amplicon (as per coordinates in Data set S1) was determined using *samtools depth* and *samtools mpileup* (minimum number of reads per nucleotide set to 8), respectively. Different parameter combinations were tested in order to evaluate the effect on the final results. Variant SNPs were called and filtered using VarScan2 (v2.3.9) (6) with option *pileup2snp* with minimum depth of 40-100-200-400 reads, and minimum frequency

of 50% and 80%. Additional analyses were performed on the frequency of 80% set. Statistical data analysis and sequence quality on the *FAST5*, *FASTQ* and *BAM* files, including read length histograms, cumulative yield plots, quality scores, reference identity, read mapping quality, error rate (percent identity), were conducted using NanoPack (v1.0.1) (7), AlignQC (v2.0.5) and Qualimap2 (v2.2.1) tools for the nanopore aligned fastq. Finally, Integrative Genomics Viewer (v2.4.14) (IGV, Broad Institute, Boston, MA) was used to visualize alignment files. Read depth and MinION-MiniSeq variant calling comparison plots were generated using seaborn/pandas/matplotlib modules in python.

*MiniSeq*

The Deeplex Myc-TB Web Application 1.0.3 (https://deeplex.bluebee.com/deeplex) provided by the manufacturer was used to analyze the FASTQ files obtained from the MiniSeq sequencing, reporting taxonomic information, sequencing depth, and variant-based resistotype. The MTBseq pipeline was also applied to generate comparison analyses with MinION. The error rate for Illumina fastq was calculate using BioAlcidaeJdk tool (8) as reported on http://lindenb.github.io/jvarkit/BioAlcidaeJdk.html ("*percent identity from bam file").*

## Results

### MiniSeq run quality

The study collection was batched on four sequencing runs on a MiniSeq instrument to serve as the reference standard sequencing. Percentages of clusters passing filter (PF) and Phred-like quality scores of 30 (Q30) were 93.2, 89.3, 91.0, 86.1 and 91.3, 90.5, 89.7, 87.7 for the four runs, respectively. Sequence length of MiniSeq data was 2x150 base pairs. The average depth of sequencing coverage obtained on MiniSeq was 4177x with

median values over 4000x and standard deviation based on the entire dataset of 1578x (Data set S2). Minimum depth of coverage of the study samples was 1102x.

**MinION runs quality**

The MinION pore occupancy (512 active channels available at the beginning for both runs) was ≥ 70% of active pores for both runs, indicating good quality libraries were generated and runs had high data throughput. The average base call quality score of the reads was 8.9 (9.1 and 8.6 per run, respectively). More than 85% of generated reads had quality scores of >7 (default cut-off), defined by ONT as minimum acceptable quality for “passing” reads (Fig. S1). The mean MinION read length was 792 bases, reflecting the average Deeplex Myc-TB amplicon size distribution that was sequenced.

The average depth of sequencing coverage relative to reference genome obtained on MinION was 4151x with median values over 4000x and standard deviation based on the entire dataset of 1399x (Data set S2). Minimum depth of coverage of the study samples was 407x on MinION allowing for SNP calls on all amplicons for all targets.

**Variant calling: MinION *vs* MiniSeq**

Adopting a heuristic approach for MinION variant calling, we set different minimum coverage thresholds to explore variant calling at 80% minimum frequency, namely 40x, 100x, 200x and 400x, representing 1 to 10% of the coverage depth obtained on average from the sequenced samples (i.e. 4000x) (Data sets S8 to S16). The SNPs from MiniSeq runs were called by the GenoScreen Web Application, which was used to generate the bar plot of Figure 3 (purple bar). It is important to note that this application only considers the variants with at least 200x of coverage, in comparison to the MinION calls, which were generated using the different thresholds we set (above).

## References


1. Tagliani E, Alagna R, Tafaj S, Hafizi H, Cirillo DM. 2017. Evaluation of Mycobacterium tuberculosis viability in OMNIgene-SPUTUM reagent upon multi-day transport at ambient temperature. BMC Infect Dis 17:663.
2. Makhado NA, Matabane E, Faccin M, Pinçon C, Jouet A, Boutachkourt F, Goeminne L, Gaudin C, Maphalala G, Beckert P, Niemann S, Delvenne JC, Delmée M, Razwiedani L, Nchabeleng M, Supply P, de Jong BC, André E. 2018. Outbreak of multidrug-resistant tuberculosis in South Africa undetected by WHO-endorsed commercial tests: an observational study. Lancet Infect Dis 18:1350-1359.
3. Genoscreen. 2019. Deeplex Myc-TB Technical Note, V05: June 2019.
4. Wick RR, Judd LM, Gorrie CL, Holt KE. 2017. Completing bacterial genome assemblies with multiplex MinION sequencing. Microb Genom 3:e000132.
5. Li H. 2018. Minimap2: pairwise alignment for nucleotide sequences. Bioinformatics 34:3094-3100.
6. Koboldt DC, Zhang Q, Larson DE, Shen D, McLellan MD, Lin L, Miller CA, Mardis ER, Ding L, Wilson RK. 2012. VarScan 2: somatic mutation and copy number alteration discovery in cancer by exome sequencing. Genome Res 22:568-76.
7. De Coster W, D'Hert S, Schultz DT, Cruts M, Van Broeckhoven C. 2018. NanoPack: visualizing and processing long-read sequencing data. Bioinformatics 34:2666-2669.
8. Lindenbaum P, Redon R. 2018. bioalcidae, samjs and vcffilterjs: object-oriented formatters and filters for bioinformatics files. Bioinformatics 34:1224-1225.

**Supplementary Figure**

Fig. S1: Average read quality as function of mean read lengths of the two MinION runs (48 and 56 samples, respectively).

Run 48 samples

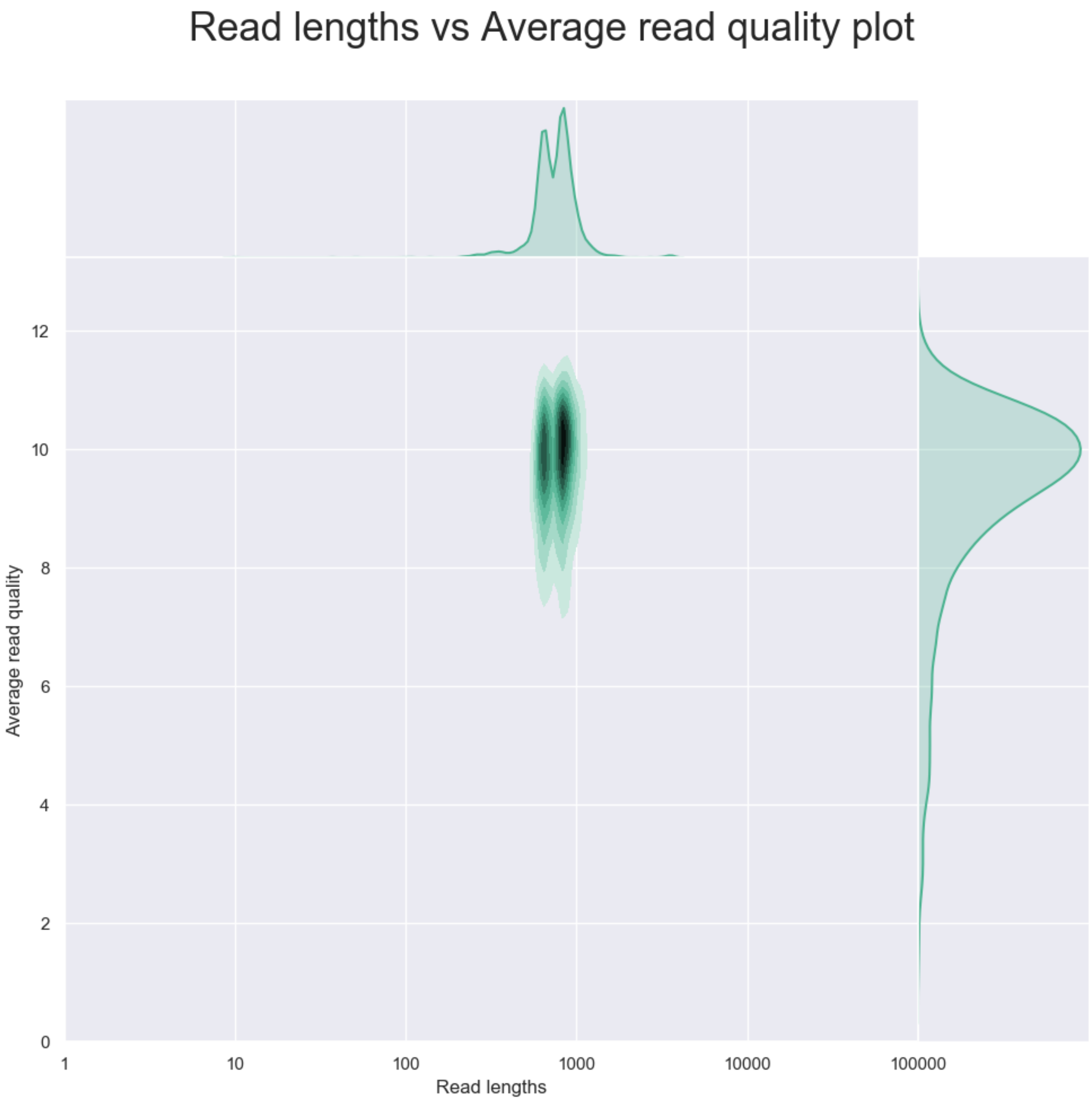

Run 56 samples

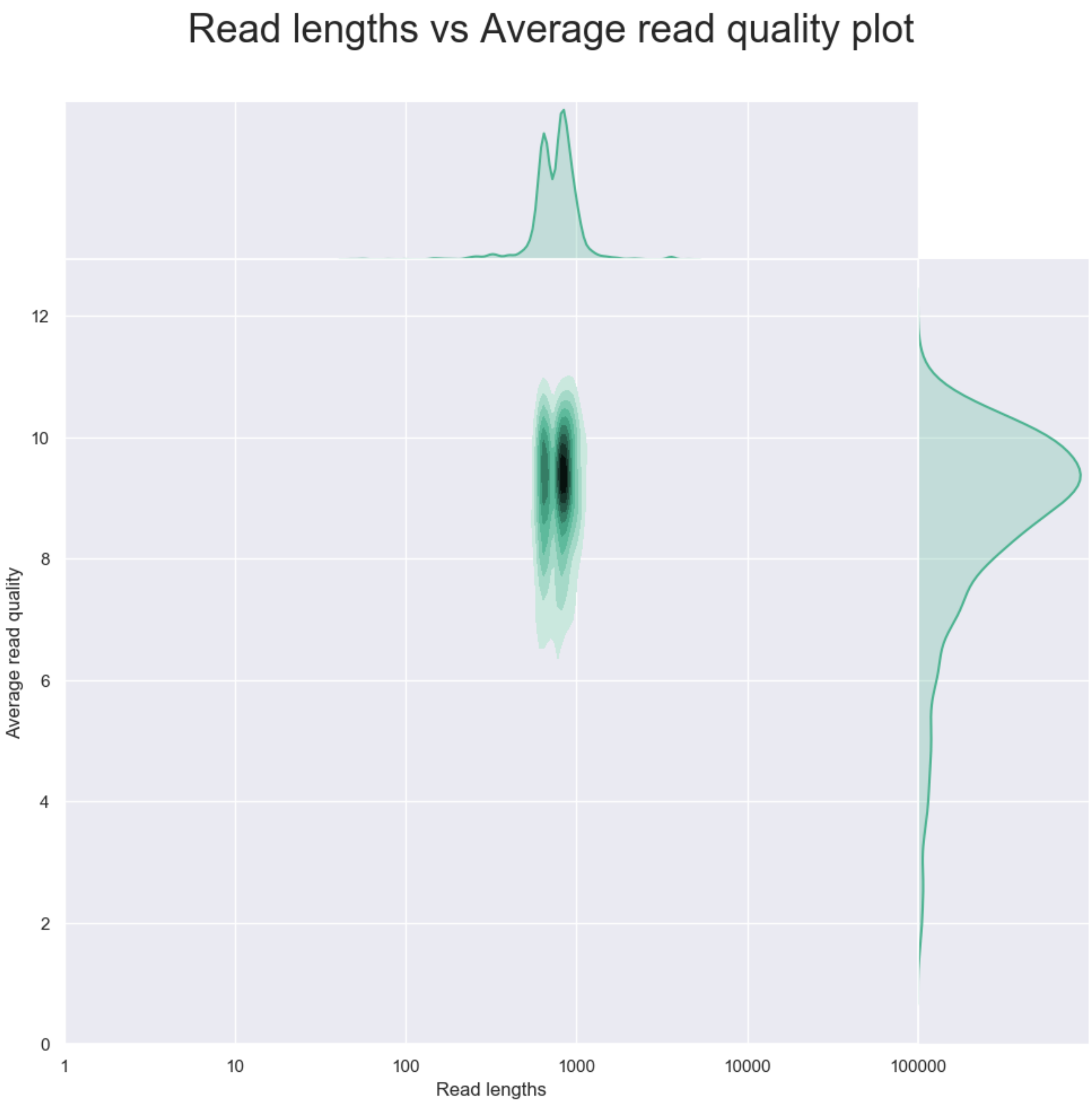

Supplement: Supplemental file 1 [file JCM.00632-20-s0001.pdf]
